# Characterization of Methicillin-Resistant *Staphylococcus aureus* Isolates from Periprosthetic Joint Infections

**DOI:** 10.3390/pathogens11070719

**Published:** 2022-06-23

**Authors:** Jiun-Liang Chen, Tsung-Yu Huang, Wei-Bin Hsu, Chiang-Wen Lee, Yao-Chang Chiang, Pey-Jium Chang, Kuo-Ti Peng

**Affiliations:** 1 Department of Orthopaedic Surgery, Chang Gung Memorial Hospital, Chiayi 61363, Taiwan; yq0139@cgmh.org.tw; 2 College of Medicine, Chang Gung University, Taoyuan 33303, Taiwan; r12045@cgmh.org.tw; 3 Division of Infectious Diseases, Department of Internal Medicine, Chang Gung Memorial Hospital, Chiayi 61363, Taiwan; 4 Department of Nursing, Chang Gung University of Science and Technology, Chiayi Campus, Chiayi 61363, Taiwan; 5 Sports Medicine Center, Chang Gung Memorial Hospital, Chiayi 61363, Taiwan; wbhsu@cgmh.org.tw; 6 Department of Nursing, Division of Basic Medical Sciences, Recurrent Diseases and Health Promotion Research Center, Chang Gung University of Science and Technology, Puzi City 61363, Taiwan; cwlee@mail.cgust.edu.tw (C.-W.L.); yaochang.chiang@gmail.com (Y.-C.C.); 7 Research Center for Industry of Human Ecology and Research Center for Chinese Herbal Medicine, Chang Gung University of Science and Technology, Guishan Dist., Taoyuan 33303, Taiwan; 8 Department of Rehabilitation, Chang Gung Memorial Hospital, Chiayi 61363, Taiwan; 9 Graduate Institute of Clinical Medical Sciences, College of Medicine, Chang-Gung University, Taoyuan 33302, Taiwan; peyjiumc@mail.cgu.edu.tw; 10 Department of Nephrology, Chang Gung Memorial Hospital, Chiayi 61363, Taiwan

**Keywords:** periprosthetic joint infection, methicillin-resistant *Staphylococcus aureus*, genotypes, phenotypes

## Abstract

Periprosthetic joint infection (PJI) is a troublesome clinical issue in total joint arthroplasty (TJA). Although methicillin-resistant *Staphylococcus aureus* (MRSA) is considered to be the most serious pathogen in PJIs, little is known about the genotypic and phenotypic characteristics of MRSA clones isolated from PJI patients. A total of 36 MRSA isolates from PJI patients were collected at the Chang-Gung Memorial Hospital in Taiwan from May 2016 to October 2019. All MRSA isolates were subjected to genome typing. The prevalence of Panton–Valentine leucocidin (PVL), the antibiotic susceptibility profile, and the biofilm formation ability were compared among different MRSA genogroups. Additionally, demographics and clinical manifestations of patients infected with different MRSA genogroups were investigated. Eight sequence types (STs) were identified among 36 isolated from PJIs. According to the incidence of MRSA genotypes in PJIs, in this study, we divided them into four groups, including ST8 (*n* = 10), ST59 (*n* = 8), ST239 (*n* = 11), and other STs (*n* = 7). For the antibiotic susceptibility testing, we found that all MRSA isolates in the ST239 group were highly resistant to ciprofloxacin, gentamicin trimethoprim-sulfamethoxazole, and levofloxacin. Additionally, ST239 MRSA also had a higher ability to form biofilm than other groups. Importantly, patients with ST239 infection typically had a fever and exhibited higher levels of inflammatory markers, including C-reactive protein (CRP) and white blood cell count (WBC). Epidemiological investigations revealed that knee PJIs were mainly attributed to infection with ST59 MRSA and increasing trends for infection with ST8 and other ST types of MRSAs in PJI patients were observed from 2016 to 2019. The identification of MRSA genotypes in PJIs may be helpful for the management of PJIs.

## 1. Introduction

Total joint arthroplasty (TJA) is one of the most common orthopaedic surgeries, and the number of patients undergoing TJA is gradually growing [1,2,3]. Periprosthetic joint infection (PJI) is a devastating complication occurring in 0.5% to 3% of primary and in 4–6% of revision arthroplasties [4] and associated with fateful morbidity and mortality. Moreover, the treatment failure rate of PJIs is still high, over 30% for surgical debridement with implant retention or 10~20% for one- and two-stage exchange arthroplasty [1,5,6,7,8,9,10].

*Staphylococcus aureus* (*S. aureus*), a Gram-positive bacterium, is a major human pathogen isolated from PJIs [10,11]. The proportion of methicillin-resistance *Staphylococcus aureus* (MRSA) gradually increases in clinical isolates of *S. aureus* [12], even over 50% prevalence among hospital pathogens [13]. Several factors, including multi-antibiotic resistance, biofilm, etc., make MRSA difficult to eradicate, resulting in several serious health care issues [14]. Meanwhile, various MRSA strains have different antibiotic resistances, mainly depending on the carry of antibiotic-resistance genes and biofilm-forming abilities [15]. Hence, identifying the MRSA strain and selecting adequate treatment strategies to predict the prognosis are important issues in PJIs for orthopaedists; however, there is still a lack of information surrounding these issues in PJIs. In this study, we aimed to use molecular methods to identify the MRSA strains isolated from PJIs and further link their genotypic to the phenotypes of antibiotic profiles, biofilm formation rate, and clinical presentation to draw the whole relationship. We hypothesize that this information could be a reference for orthopaedists to give adequate treatment strategies for treating MRSA-infected PJIs.

## 2. Results

### 2.1. Demographic and Clinical Characteristics of Patients with MRSA-Induced PJIs

A total of 36 MRSA isolates from PJI patients were included in this study. The numbers of male and female PJI patients were 20 (56%) and 16 (44%), respectively (Table 1), and the mean age of these patients was 64 years old (ranging from 26 to 98 years old). The hip (81%) and knee (17%) were the most common infection sites. In these PJI patients, the most prevalent underlying diseases were chronic hepatitis and cirrhosis (CHC, 47%) and diabetes mellitus (DM, 25%).

### 2.2. Molecular Typing of MRSA Isolates from PJIs

Table 2 shows the molecular genotyping analyses of MRSA isolates from PJI patients. By the SCC*mec* typing, these MRSA isolates showed that the most prevalent genotypes were both SCC*mec* type III (36%) and type IV (36%), followed by SCC*mec* type V (25%). Eight distinct STs were obtained from the MLST analysis in these MRSA isolates. Among these sequence types, the most prevalent STs were ST239 (31%, *n* = 11), ST8 (28%, *n* = 10), and ST59 (22%, *n* = 8). The analysis of the polymorphic X region of the *spa* gene revealed that nine *spa* types were found within the MRSA isolates, and the top three *spa* types were t037 (31%), t008 (25%), and t437 (22%). In these MRSA isolates, we also found that 61% (*n* = 22) were positive for PVL toxin genes (Table 2). We mainly regrouped this classification of the MRSA genotype based on the results of MLST analysis shown in Table 3. Among them, the most prevalent lineages were ST239-t037-SCC*mec*III (*n* = 11, 31%), followed by ST8-t008-SCC*mec*IV (*n* = 7, 19%), and ST59-t437-SCC*mec*V (*n* = 5, 14%). To attempt to compare the characteristics of various MLST genotypes, we divided these MRSA isolates into four groups: ST8 (*n* = 10), ST59 (*n* = 8), ST239 (*n* = 11), and other ST (*n* = 7). Noteworthily, the prevalence rate of PVL in the ST8 group (9/10, 90%) was remarkably higher than that in the ST59 (6/8, 75%), ST239 (5/11, 45%), and other ST (2/7, 29%) groups (Table 3).

### 2.3. Association of Antibiotic Resistance and Genotypes of MRSA in PJIs

The relationships of the antibiotic resistance and MLST-classified MRSA in PJIs are shown in Table 4. The lowest concentration of an antibiotic that completely inhibits bacterial growth is defined as the minimum inhibitory concentration (MIC). Table 5 shows the value of each strain for all antibiotics. These MRSA isolates from PJIs exhibited high rates of resistance to ciprofloxacin (67%, *n* = 24), gentamicin (44%, *n* = 17), and TMP-SMX (31%, *n* = 11). However, the rates of resistance to fusidic acid and rifampicin for these MRSA isolates were 14% (*n* = 5), and 6% (*n* = 2), respectively. When the antibiotic susceptibility profiles were compared among the MRSA groups, we found that the antibiotic-resistant patterns varied among these groups (Table 4). The results showed that ST239 isolates exhibited one hundred percent resistance to ciprofloxacin, gentamicin, and TMP-SMX; however, the high rates of antibiotic resistance were not generally observed in the ST8, ST59, and other ST isolates. Noteworthily, TMP-SMX resistance only occurred in the ST239 MRSA isolates (*n* = 11, 100%), and rifampicin resistance was found only in the other ST isolates (ST5, *n* = 2).

**Figure 1:**
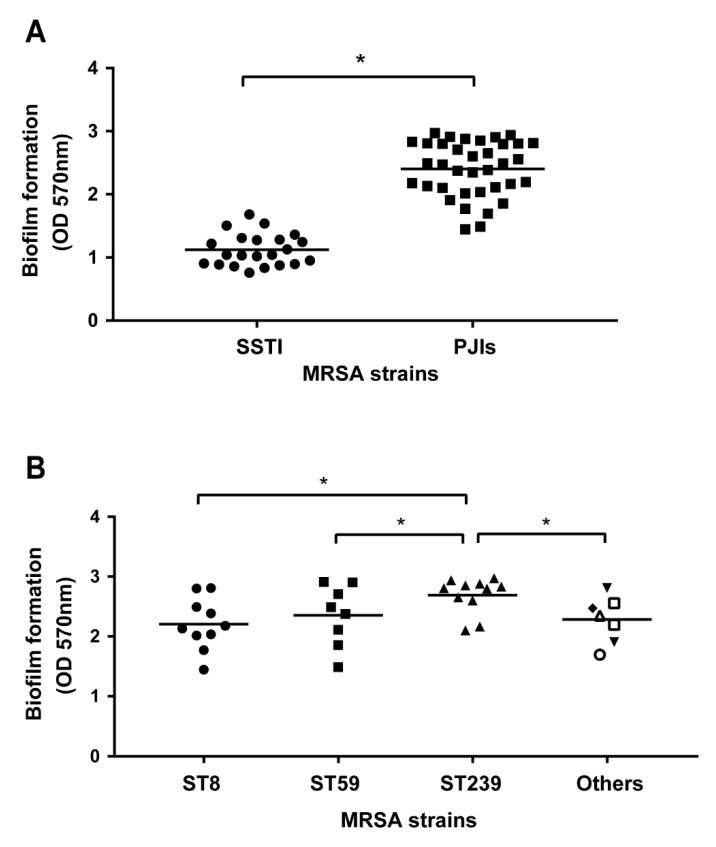
Biofilm formation of MRSA clinical isolates. The biofilm formation of MRSA isolates was investigated after 24 h incubation in a microtiter plate: (**A**) Comparison of biofilm formation capacities of MRSA from the skin and soft tissue infection (SSTI) (*n* = 22) and periprosthetic joint infection (PJIs) (*n* = 36). Each shape (● and ■) represents individual isolates, and bars indicate the means, * *p* < 0.05 are considered significant; (**B**) biofilm formation of PJIs-MRSA clinical isolates with different ST types. Each shape (●, ■, ▲, ▼, ◆, ○, □, and △) represents individual isolates categorized into different ST types, and bars indicate the means, * *p* < 0.05 is considered to be significant. In other ST strains, the shapes ▼, ◆, ○, □, and △ represents ST5, ST7, ST45, ST508, ST1232, respectively.

Although all MRSA isolates from PJIs were sensitive to vancomycin (MIC < 2 μg/mL), some MRSA isolates were found with reduced vancomycin susceptibility (MIC = 1.5 μg/mL) (data not shown). Therefore, we attempted to analyze and compare vancomycin MICs in different MRSA groups (Figure 1). Our results revealed that both the ST59 and ST239 isolates had higher MICs of vancomycin than the ST8 and other ST isolates (Supplementary Figure S1).

### 2.4. Association of Biofilm Formation and Genotypes of MRSA in PJIs

The biofilm formation ability is one of critical factors of MRSA virulence. Thus, the biofilm formation ability of PJI-isolated MRSA isolates was assessed and compared (Figure 1). The results showed the ST239 isolates exhibited the highest capacity for biofilm formation ability (Figure 1). As compared with MRSA types isolated from skin and soft tissue infection (SSTI) patients, the PJI-isolated MRSA isolates had a greater biofilm-forming ability (Supplementary Figure S1, *p* < 0.0001), suggesting that PJI-isolated MRSAs may generally have a better ability for colonizing the host and resisting the clearance by the host than SSTI-isolated MRSA types.

### 2.5. Correlation between MRSA Genotypes and Clinical Phenotypes

**Figure 2:**
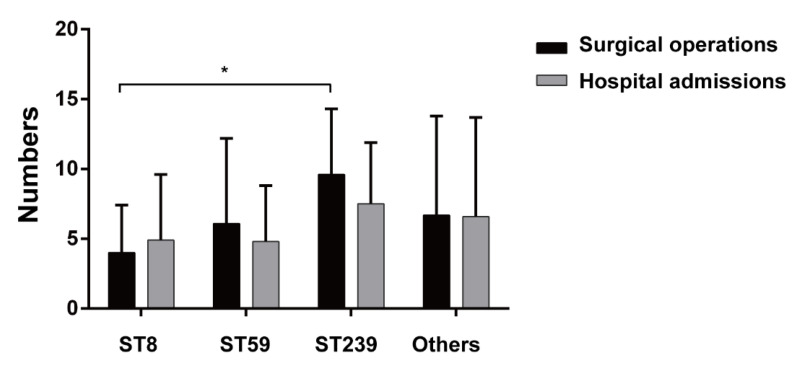
Numbers of operations and admissions in PJIs. The medical record review and analysis showed that the number of surgical debridements and the number of hospital admissions were generally high for PJI patients with ST239 MRSA infection as compared with infections with the other MRSA genogroups. Data are expressed as means ± SD, * *p* < 0.05 is considered to be significant.

To further explore the potential relationship between MRSA genotypes and clinical features, the disease phenotypes or severity in PJI patients were analyzed using data from patient records. A medical record review and analysis showed that the number of surgical debridements and the number of hospital admissions were generally high for PJI patients with ST239-type MRSA infection as compared with the other ST types of MRSA (Figure 2). These results implicated that ST239-type MRSA infection could cause more refractory cases than infection with the other ST types of MRSA in PJI patients.

**Figure 3:**
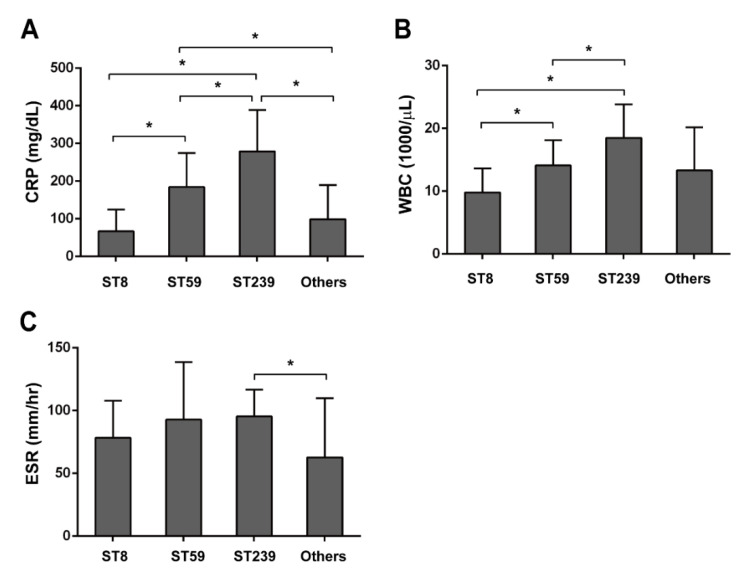
The association of biomarkers of pre-operative status and MRSA strains: (**A**) C-reactive protein value (CRP); (**B**) white blood cell count (WBC); (**C**) erythrocyte sedimentation rate (ESR) were measured. Data are expressed as means ± SD, * *p* < 0.05 is considered to be significant.

Moreover, we also assessed and compared different inflammatory markers, including C-reactive protein (CRP), white blood cell count (WBC), and erythrocyte sedimentation rate (ESR) in these PJI patients. As shown in Table 5, we found that the serum levels of CRP and WBC were also significantly higher in the ST239 types than in other ST types (Figure 3A,B). However, there was no evident difference in ESR levels between these different ST types (Figure 3C). According to the medical records, we additionally found that the incidence of fever was remarkably higher in ST239-induced PJI patients (7 out of 11 cases) than other ST-induced PJI patients (Table 6).

### 2.6. Epidemiological Characteristics of MRSA-Induced PJIs

**Figure 4:**
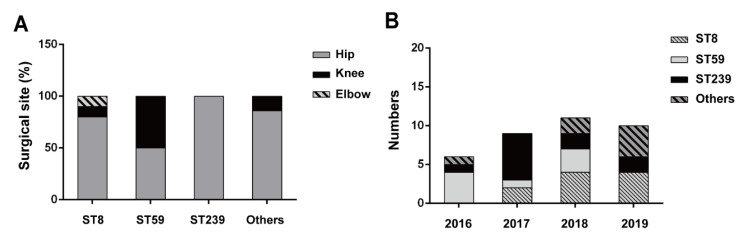
The surgical site and annual trends of MRSA strains: (**A**) The percentage of the surgical sites; (**B**) yearly cases numbers of ST8, ST59, ST239, and other ST MRSA in PJI patients.

We regrouped the patient’s demographics by different ST types of MRSA. After analysis, we found eighty percent of the ST8-type MRSA in the men’s cases (*n* = 8), whereas the average age of MRSA infection seemed to be younger in the ST239 type (Table 7). Particularly, we noticed that most of the ST239 infections in PJIs (nine out of eleven cases) occurred in chronic hepatitis and cirrhosis (CHC) patients (Table 6). For analysis of infection sites, although there were only six cases of knee PJI included in the study (Table 1), we found that these knee PJIs were mainly caused by ST59-type MRSA (four out of six cases) (Figure 4A). It is also worth noting that all ST239-type MRSA (*n* = 11) were found only in hip PJIs, but not in knee or elbow PJIs (Figure 4A). Additionally, according to the collection years, the temporal distribution of MRSA genotypes in PJIs was determined in the study. As shown in Figure 4B, increasing trends for the ST8 and other ST types were observed in PJI patients from 2016 to 2019.

## 3. Discussion

In our study, the ST239-type MRSA had a higher multidrug-resistance (resistant to ciprofloxacin, gentamicin, TMP-SMX, levofloxacin) (Table 4) and biofilm-forming ability (Figure 2) than other types of MRSA in PJIs, suggesting that ST239-type MRSA might have a higher virulence. Additionally, ST239-type MRSA made PJI patients have a fever and higher inflammatory markers (Table 5 and Figure 3) among MRSA types. These results might associate with enterotoxins because these can be superantigens, and therefore, activate toll-like receptors (TLR) causing increased inflammatory response. We analyzed the relationship between PVL and MLST, and PVL is one of the enterotoxin and an important factor in inflammation response. (Table 2) For future research, it would be important to analyze the profile of other enterotoxin genes. Notably, ST239-type MRSA isolates (*n* = 11) were deep infectious because all ST239-type MRSA were collected from hip PJIs, but not in knee or elbow (Figure 4A). It was indicated that PJIs with ST239-type MRSA would be more difficult to treat among different types of MRSA (ST5, ST7, ST8, ST45, ST59, ST508, and ST1232). Fortunately, the ST239-type MRSA was still susceptible to vancomycin although the MIC value was near the defined borderline (Table 4).

Previous studies have shown that over 90% of MRSA PJIs were suitably treated with vancomycin after removal of prosthesis and radical debridement [16]. However, for MRSA PJIs with retained hardware, treatment would combine vancomycin and rifampicin together [16]. Rifampicin is an effective antibiotic, but it is notably that rifampicin should be used in combination with other antibiotics because of its rapid emergence of resistance [17]. In general, the duration of antibiotics depends on the surgical intervention conducted. Antibiotics have been used with two-stage revision for 6 weeks, one-stage revision for 12 weeks, and DAIR for 6 months to a year [9,18]; the optimal treatment condition is still uncertain [19,20]. Rifampicin, fusidic acid, TMP-SMX, doxycycline, levofloxacin, and ciprofloxacin are oral antibiotics and have been used in MRSA-infected PJIs or antibiotic suppressive therapy [19,21,22]. The results in this current study support that all ST-type MRSA were less resistant to rifampicin, doxycycline, and fusidic acid (Table 4), indicating that the rifampicin combined with fusidic acid or doxycycline may be better antibiotic choices applied to most MRSA types in PJIs. Recently, a review article indicated that the fluoroquinolones (such as ciprofloxacin and levofloxacin) are the best-documented combination partners for rifampicin [17]. The present study revealed that ciprofloxacin had high drug-resistant rates (24 in 36 isolates, 66.7%) to different types of MRSA in the PJI patients. Although the article suggests that fluoroquinolones are an excellent combination antibiotic for rifampicin, this combination strategy may not be suitable for our collected MRSA from PJIs, especially the ST239-type infective PJI patients (100% resistance rate for ciprofloxacin). In addition, vancomycin is the most important and useful drug to treat MRSA in PJIs. In this study, the MIC of vancomycin in all isolated PJI MRSA did not reach the defined value (2 µg/mL), but ST59- and ST239-type MRSA had higher MIC of vancomycin than other strains and the MIC values were about 1.5 µg/mL close to the defined value (2 µg/mL) (Supplementary Figure S1), implying that ST59- and ST239-type MRSA need higher vancomycin concentration than other types of MRSA strains in treating PJIs.

The destruction of bacteria enclosed within a self-produced matrix embedded in biofilms is very difficult. Biofilm infections on orthopedic implants and surgeries often cause serious health care concerns based on their antibiotic resistance. Unlike other surface-infected diseases, PJIs require long-term suppressive treatment by antibiotics against adherent bacteria [23]. Thus, the biofilm formation ability of MRSA should be a considerable factor in the antibiotic treatment of PJIs. Our data revealed that deep infection of the MRSA-induced PJI group indeed had significantly high biofilm formation ability as compared with the superficial MRSA-induced SSTI group (Figure 1). Meanwhile, a higher biofilm formation ability was obtained in the ST239-type MRSA as compared with other ST types (Figure 1). It might be a possible reason to explain a higher frequency of surgical operations and hospital admissions in ST239-type MRSA-infected PJIs (Figure 2). Our results echoed the previous study that reported ST239-type MRSA in early post-operative orthopedic implant-based infections has a higher biofilm formation rate as compared with other ST types (including ST22 and ST772) in India [24]. Based on our current results and previous findings [16,23], a prolong high-dose vancomycin administration might be considered.

All types of MRSA isolates from PJIs were further linked to medical records. CRP, WBC, and ESR are inflammation-related biomarkers, including bacteria-caused infection. The CRP and ESR are the two most commonly published serum biomarkers and first-line screening tests in PJIs [25]. Regarding the means of inflammation-related biomarkers of pre-operative PJI patients, the intensity of expression levels of CRP and WBC were ST239, ST59, and then other ST and ST8 types (Table 6 and Figure 3). The ESR in the ST239 type was significantly higher than other ST-type MRSA. Interestingly, a noticeable symptom of pre-operative fever was observed in ST239.

High-dose continuous intravenous vancomycin infusion was suggested for increasing the treatment success rate of hip PJIs in a previous study [18]. In the current study, all ST239-type MRSA isolates were obtained from hip PJIs, while ST59-type isolates showed 50% hip and 50% knee; ST8-type isolates showed 80% hip, 10% knee, and 10% elbow. The high biofilm formation rate, antibiotics resistance profiles, and MIC of vancomycin of ST239-type MRSA may be responsible for their deep tissue infection ability. Although the ST239 MRSA strain has been replaced by other types of MRSA strains in several countries [26], it still shows a higher population in PJIs in Taiwan. However, the trends of numbers of the cases seemed to decrease year by year (Figure 4A). Similar to the trends of ST59 in PJIs, the number of cases have gradually declined, and no ST59 infective patient was found in 2019 (Figure 4B). However, the infective trends of ST8 and other ST types notably increased in PJIs in Taiwan. The SCC*mec*IV-ST8 MRSA is majorly associated with SSTI in Japan [27] and Taiwan in recent years [28,29]. Interestingly, our results showed that the prevalence rate of PVL in the ST8-type isolates (9/10, 90%) was remarkably higher than the other three groups (Table 3). Since the majority of PVL-positive strains represent community isolates [30], our results may indicate that ST8 has penetrated the healthcare system and has deep tissue infection abilities. In other ST isolates, ST5 showed considerable resistance to multi-antibiotics as compared with other ST types. SCC*mec*IV-ST508, a single locus variant of ST45, is a common nasal carriage clone of methicillin-susceptible *S. aureus* (MSSA), but rarely as MRSA [31,32]. Recently, ST45 and ST508 MRSA have emerged in nasal and oral regions [33,34] and were also isolated from joints of the PJI patients in this study. Significantly, the SCC*mec*V-ST1232 MRSA strain, an animal-or animal contact-related strain and rarely found infection in humans [35,36], was observed in the current PJI study. There is a need to pay more attention to the further spread and profiles of antibiotic resistance of these minor ST MRSA strains in PJIs.

Limitations of this study include the small case numbers, which may not reflect the reality of the global distribution of MRSA strains and epidemic trends in PJIs. PJI cases are continuing to be collected for resolving this limitation in the future. In addition, the results of the MIC assay and biofilm formation are partially but not fully correlated to clinic records, which is an inherent limitation of retrospective research. This is just the beginning to characterize molecular genotyping of methicillin-resistant *Staphylococcus aureus* isolates in 36 PJI cases. In addition, we plan to analyze the profile and pattern of enterotoxin gene and virulence factors in our future research. Our findings provide the relationships of genotypic and clinic phenotypic MRSA strains and a suggested antibiotics management strategy in PJIs, which may not be directly generalized to other countries or regions with different treatment strategies or insurance rules.

## 4. Materials and Methods

### 4.1. Bacterial Isolates and Patient Characteristics

A total of 36 MRSA isolates were collected from acute PJI patients from May 2016 to October 2019. The Institutional Review Board of the Chang Gung Medical Foundation approved the retrospective study (number 202001374B0). Information was collected from an electronic medical records review and recorded on the MRSA database. Data obtained regarding the study subjects included basic demographics, medical history (underlying diseases), MRSA infection sites, surgical debridement, and number of hospital admissions. The means of pre-operative biomarkers, including C-reactive protein value (CRP), erythrocyte sedimentation rate (ESR), and white blood cell count (vWBC), with fever (>37.5 °C) or not, were recorded and analyzed.

### 4.2. Genomic DNA Extraction

The MRSA isolates were inoculated in tryptic soy broth (TSB) overnight, and 1 mL of overnight culture product was harvested by centrifugation. DNA was extracted from the bacterial pellet using a Mini gDNA bacteria Kit (Geneaid, Taipei, Taiwan) with lysostaphin, according to the manufacturer’s instructions.

### 4.3. SCCmec Typing, Multilocus Sequence Typing (MLST), and Spa Typing

Several molecular methods have been developed for the classification of MRSA types, including the staphylococcal cassette chromosome *mac* (SCC*mec*), the X region encoding protein A (*spa*), Panton–Valentine leukocidin (*pvl*), and multilocus sequence typing (MLST) [37,38,39]. Multiplex PCR with four primer pairs was performed to identify the SCC*mec* types I–V [29]. Multilocus sequence typing (MLST) was carried out by PCR and sequencing of the seven housekeeping genes, carbamate kinase (*arcC*), shikimate dehydrogenase (*aroE*), glycerol kinase (*glp*), guanylate kinase (*gmk*), phosphate acetyltransferase (*pta*), triosephosphate isomerase (*tpi*), and acetyl coenzyme A acetyltransferase (*yqiL*). The allelic number and profile (sequence type or ST) of each gene was determined by the *S. aureus* MLST database (http://saureus.mlst.net/, accessed on 16 July 2021). The staphylococcal protein A gene polymorphic region (*spa*) of each MRSA isolate was amplified by the PCR method, and the amplified products were sequenced. The variable repeat region of the *spa* gene was analyzed using the Ridom StaphType software (version 2.2.1; Ridom, GmbH, Wurzburg, Germany) [29].

### 4.4. Detection of Panton–Valentine Leukocidin (pvl)

Amplification of the *pvl* gene was done with primers luk-PV-1 and luk-PV-2, as described in our previous study [29].

### 4.5. Antibiotic Susceptibility Test

The minimum inhibitory concentration (MIC) of fusidic acid, trimethoprim-sulfamethoxazole (TMP-SMX), rifampicin, ciprofloxacin, gentamicin, and vancomycin were determined using the E-test method, according to the manufacturer’s guidelines. Inoculates were adjusted to a 0.5 McFarland turbidity standard (10^8^ CFU/mL) and plated onto Mueller–Hinton agar. E-test gradient strips (Etest^®^, bioMérieux SA, France) were applied to the inoculated agar surface. The plates were incubated at 37 °C for 18 h. The MIC was read at the point where the zone of inhibition intersected the antimicrobial agent concentration scale on the strip. The thresholds of MIC results were chosen following the guidelines from the Clinical Laboratory Standards Institute (CLSI) and our previous studies [29,40].

### 4.6. Biofilm Formation Assay

To evaluate the biofilm formation capacity of MRSA isolates from PJIs, we also included another 22 MRSA isolates from patients with skin and soft tissue infection (SSTI) during the same period. Surgical debridement for servility of wound infection was performed for all SSTI patients. There were no age and gender differences between SSTI and PJI patients (demographics of SSTI are presented in Supplementary Table S1).

The biofilm assay was conducted in a 96-well microplate. The bacterial isolates were grown in tryptic soy broth (TSB) supplemented with 0.25% glucose at 37 °C overnight. The suspension of bacteria was adjusted to achieve turbidity equivalent to a 0.5 McFarland standard. Then, aliquots of 200 μL per well were added to a 96-well microplate and incubated at 37 °C for 24 h. The negative control wells contained only broth. A known biofilm-forming *S. aureus* reference strain, ATCC 29213, was used as the positive control. After incubation, wells were gently washed with phosphate-buffered saline (PBS), fixed using 99% methanol, and dried at room temperature. The attached biofilm cells were stained with 0.1% crystal violet, and the bound dye was dissolved using 33% glacial acetic acid. The optical density (OD) of each well was measured at 570 nm using an ELISA reader.

### 4.7. Statistical Analysis

All data were analyzed using the GraphPad Prism version 7.0 (GraphPad Software Inc., San Diego, CA, USA). Student’s *t*-test and ANOVA were used to compare proportions. A *p*-value <0.05 was considered to be statistically significant.

## 5. Conclusions

In conclusion, our laboratory and clinic results revealed a higher pathogenicity of ST239 than the other ST-type MRSA strains and the resulting dissatisfaction prognosis in PJIs. The characteristics of multiple antibiotic resistance, high MIC of vancomycin, and biofilm formation ability of ST239 might lead to the higher level of inflammation biomarkers and the higher number of surgical debridements than ST59, ST8, and other ST-type isolates. However, the vancomycin MIC of ST59- and ST239-type MRSA were close to the defined value (2 µg/mL). Based on our current results and previous findings, prolong high-dose vancomycin administration might be considered.

Furthermore, due to the increasing number of ST8 and other ST types of infected cases in deep infectious sites, research efforts should focus on their future spread and drug resistance status. This is the first study to focus on PJIs in Taiwan; the results support the importance of sequencing the genotype of MRSA strains using MLST in association with clinical phenotypes as a useful method for assisting orthopaedists to better treat MRSA-induced PJIs.

## Figures and Tables

**Table 1:** Demographics of patients with MRSA PJIs.

Characteristic	PJIs (*n* = 36)
Sex	
Female	16 (44%)
Male	20 (56%)
Age (year)	
Mean ± SD	64 ± 18
Range	26–98
Surgical sites	
Elbow	1 (3%)
Hip	29 (81%)
Knee	6 (17%)
Underlying diseases	
CHC	17 (47%)
CKD	3 (8%)
DM	9 (25%)
Gout	3 (8%)
Cancer	2 (6%)

Abbreviation: MRSA—methicillin-resistant *Staphylococcus aureus*; PJIs—periprosthetic joint infections; CHC—chronic hepatitis and cirrhosis; CKD—chronic kidney disease; DM—diabetes mellitus.

**Table 2:** Molecular genotyping analysis of MRSA isolates from PJIs (*n* = 36).

SCC*mec* Type, No. (%)			MLST, No. (%)		*Spa* Type, No. (%)		PVL Positive, No. (%)	
II	1 (3%)		ST5	2 (6%)	t002	2 (6%)	22 (61%)	
III	13 (36%)		ST7	1 (3%)	t008	9 (25%)		
IV	13 (36%)		ST8	10 (28%)	t015	2 (6%)		
V	9 (25%)		ST45	1 (3%)	t034	1 (3%)		
			ST59	8 (22%)	t037	11 (31%)		
			ST239	11 (31%)	t091	1 (3%)		
			ST508	2 (6%)	t437	8 (22%)		
			ST1232	1 (3%)	t441	1 (3%)		
					t1081	1 (3%)		

Abbreviations: MRSA—methicillin-resistant *Staphylococcus aureus*; PJIs—periprosthetic joint infections; SCC*mec*—staphylococcal cassette chromosome *mec*; MLST—multilocus sequence typing; ST—sequence type; *spa*—staphylococcal protein A; PVL—Panton–Valentine leucocidin.

**Table 3:** Genogroups of MRSA isolates in PJIs (*n* = 36).

Genogroups	Lineages	MRSA Isolates, %
ST8 (*n* = 10)	t008-SCC*mec*IV-PVL+	6 (17%)
	t008-SCC*mec*IV-PVL-	1 (3%)
	t008-SCC*mec*V-PVL+	2 (6%)
	t441-SCC*mec*IV-PVL+	1 (3%)
ST59 (*n* = 8)	t437-SCC*mec*IV-PVL+	1 (3%)
	t437-SCC*mec*IV-PVL-	2 (6%)
	t437-SCC*mec*V-PVL+	5 (14%)
ST239 (*n* = 11)	t037-SCC*mec*III-PVL+	5 (14%)
	t037-SCC*mec*III-PVL-	6 (17%)
Others (*n* = 7)		
ST5	t002-SCC*mec*II-PVL-	1 (3%)
	t002-SCC*mec*III-PVL-	1 (3%)
ST7	t091-SCC*mec*III-PVL+	1 (3%)
ST45	t1081-SCC*mec*V-PVL-	1 (3%)
ST508	t015-SCC*mec*IV-PVL-	2 (6%)
ST1232	t034-SCC*mec*V-PVL+	1 (3%)

Abbreviations: MRSA—methicillin-resistant *Staphylococcus aureus*; PJI—periprosthetic joint infections; ST—sequence type; SCC*mec*—staphylococcal cassette chromosome *mec*; PVL—Panton–Valentine leucocidin.

**Table 4:** Association between antibiotic resistance profiles and genotypes of MRSA isolates from PJIs (*n* = 36).

Antibiotic	MIC ^a^ (µg/mL)	Total Strains (*n* = 36)	MRSA Genogroups (Resistance, %)							
			ST8 (*n* = 10)	ST59 (*n* = 8)	ST239 (*n* = 11)	ST5 (*n* = 2)	ST7 (*n* = 1)	ST45 (*n* = 1)	ST508 (*n* = 2)	ST1232 (*n* = 1)
Ciprofloxacin	≥4	24 (67%)	8 (80)	2 (25)	11 (100)	2 (100)	0	1 (100)	0	0
Gentamicin	≥16	17 (44%)	1 (10)	3 (38)	11(100)	2 (100)	0	0	0	0
TMP-SMX	≥4	11 (31%)	0	0	11 (100)	0	0	0	0	0
Fusidic acid	≥1	5 (14%)	1 (10)	2 (25)	1 (9)	0	1 (100)	0	0	0
Rifampicin	≥4	2 (6%)	0	0	0	2 (100)	0	0	0	0
Vancomycin	≥2	0	0	0	0	0	0	0	0	0

^a^ MIC values determined by E-test. Abbreviations: MLST—multilocus sequence typing; MRSA—methicillin-resistant *Staphylococcus aureus*; MIC—minimum inhibitoryconcentration; ST—sequence type; PJIs—periprosthetic joint infections; TMP-SMX—trimethoprim-sulfamethoxazole.

**Table 5:** The MIC of values of each strain for all antibiotics.

MLST	Strain	MICof Antibiotics (μg/mL)					
		Ciprofloxacin	Gentamicin	TMP-SMX	Fusidic Acid	Rifampicin	Vancomycin
ST8	Sta-25	12	12	0.064	3	0.008	1.5
	Sta-30	12	0.5	0.064	0.064	0.006	1
	Sta-581	32	0.38	0.064	0.047	0.006	0.5
	Sta-582	0.19	0.38	0.047	0.047	0.006	0.5
	Sta-596	0.19	0.25	0.047	0.032	0.006	0.5
	Sta-695	16	0.25	0.047	0.032	0.002	1
	Sta-1595	6	0.25	0.047	0.19	0.004	0.5
	Sta-1629	8	32	0.047	0.125	0.004	0.5
	Sta-1671	8	12	0.047	0.094	0.004	0.75
	Sta-1708	8	0.38	0.047	0.047	0.004	0.5
ST59	P2	0.25	256	0.064	3	0.008	1
	P11	0.25	16	0.094	0.032	0.016	1.5
	P16	0.25	0.38	0.032	0.064	0.008	1.5
	P65	0.25	64	0.064	0.047	0.006	1
	Sta-329	32	0.38	0.047	0.032	0.008	1.5
	Sta-414	0.25	4	0.032	0.032	0.004	0.5
	Sta-468	32	0.5	0.032	0.047	0.006	1.5
	Sta-790	0.25	8	0.047	1.5	0.004	0.38
ST239	P61	32	256	8	0.064	0.006	1.5
	Sta-271	32	256	8	0.047	0.004	1
	Sta-297	32	256	8	0.032	0.004	1
	Sta-311	32	256	12	0.032	0.004	1
	Sta-314	32	256	6	0.032	0.004	0.75
	Sta-373	32	256	4	2	0.004	1
	Sta-390	32	256	32	0.047	0.004	1.5
	Sta-436	32	256	4	0.094	0.004	1
	Sta-635	32	256	12	0.19	0.006	1
	Sta-1597	32	256	32	0.064	0.006	1.5
	Sta-1730	32	256	32	0.064	0.006	1
ST5	Sta-697	32	96	0.047	0.047	32	0.75
	Sta-1709	32	128	0.047	0.094	32	0.5
ST7	P9	0.25	12	0.064	3	0.004	1
ST45	Sta-1628	12	0.38	0.047	0.19	0.004	0.38
ST508	Sta-516	0.125	0.25	0.047	0.047	0.006	0.38
	Sta-1411	0.125	0.19	0.047	0.125	0.006	0.75
ST1232	Sta-1505	0.25	0.19	0.047	0.19	0.006	0.5

Abbreviations: MLST—multilocus sequence typing; MRSA—methicillin-resistant *Staphylococcus aureus*; MIC—minimum inhibitoryconcentration; ST—sequence type; TMP-SMX—trimethoprim-sulfamethoxazole.

**Table 6:** Levels of biomarkers of pre-operation in PJIs.

Variables	ST8 (*n* = 10)	ST59 (*n* = 8)	ST239 (*n* = 11)	ST5 (*n* = 2)	ST7 (*n* = 1)	ST45 (*n* = 1)	ST508 (*n* = 2)	ST1232 (*n* = 1)
CRP:mg/dL	66.8 ± 57.56	184.29 ± 90.60	279.16 ± 109.53	160.3 ± 0	78.52	218.92	97.54 ± 135.18	14.84
WBC:/μL	9.79 ± 3.86	14.1 ± 4.02	18.49 ± 5.32	12.8 ± 0	N.A	13.4 ± 4.02	14.6 ± 8.49	10.1
ESR:mm/hr	78.29 ± 29.53	92.75 ± 45.83	95.33 ± 21.21	86 ± 0	140	N.A	15.15 ± 5.44	60
Fever (no./total no.)	1/10	0/8	7/11	0/2	1/1	0/1	0/2	0/1

All data were presented as means ± SD (range). The average values of CRP, ESR, and WBC included all pre-operative data of each patient. Fever is defined as 37.5 degrees Celsius or above according to pre-operative records. Abbreviation: ST—sequence type; CRP—C-reactive protein; vWBC—white blood cell count; ESR—erythrocyte sedimentation rate; N.A.—not available, PJIs—periprosthetic joint infections.

**Table 7:** Demographics of patients in MRSA induced PJIs.

Characteristic	MRSA Isolates			
	ST8 (*n* = 10)	ST59 (*n* = 8)	ST239 (*n* = 11)	Others (*n* = 7)
Sex				
Female	2 (20%)	4 (50%)	7 (64%)	3 (43%)
Male	8 (80%)	4 (50%)	4 (36%)	4 (57%)
Age (year)				
Mean ± SD	62 ± 19	70 ± 17	56 ± 16	71 ± 17
Range	26–86	40–86	29–98	41–87
Underlying diseases				
CHC	4 (40%)	2 (25%)	9 (82%)	2 (29%)
CKD	0	0	1 (9%)	2 (29%)
DM	2 (20%)	3 (38%)	1 (9%)	3 (43%)
Gout	0	2 (25%)	0	1 (14%)
Cancer	0	0	2 (18%)	0

Abbreviation: MRSA—methicillin-resistant *Staphylococcus aureus*; PJIs—periprosthetic joint infections; CHC—chronic hepatitis and cirrhosis; CKD—chronic kidney disease; DM—diabetes mellitus.

## Data Availability

Not applicable.

## Supplementary Materials

The following supporting information can be downloaded at: https://www.mdpi.com/article/10.3390/pathogens11070719/s1, Figure S1: MIC of vancomycin for MRSA isolates; Table S1: Demographics of patients with SSTI-MRSA isolates.
