# Supplementary material for: Characterization of Methicillin-Resistant Staphylococcus aureus Isolates from Periprosthetic Joint Infections
Source: Pathogens. 2022 Jun 23;11(7):719. doi: 10.3390/pathogens11070719 (PMC9316792; doi:10.3390/pathogens11070719)
Supplement: Supplementary file 1 [file pathogens-11-00719-s001.zip › pathogens-1711003-supplementary.pdf]

Supplementary Materials

Table S1. Demographics of patients with SSTI-MRSA isolates.

Characteristic		SSTI-MRSA (n = 22)
Sex		
	Female	14 (64%)
	Male	8 (36%)
Age (year)		
	Mean ± SD	53 ± 20
	Range	9–82
Infected sites		
	Head and neck	10 (45%)
	Trunk	6 (27%)
	Limbs	6 (27%)

Abbreviation: SSTI—skin and soft tissue infection; MRSA—methicillin-resistant *Staphylococcus aureus*.

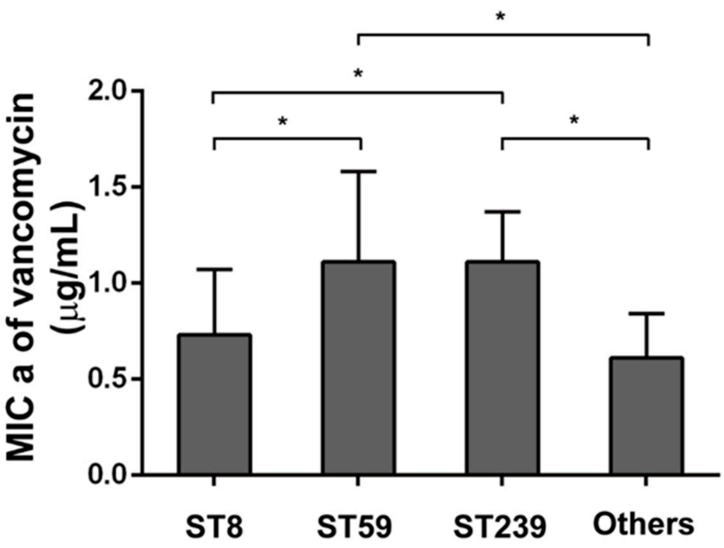

Figure S1. MIC of vancomycin for MRSA isolates. The minimum inhibitory concentration (MIC) of vancomycin was determined with the E-test method. Compared to ST8 and other strains groups, the ST239 and ST59 groups had a highly significant MIC in treating PJIs with vancomycin, *$p < 0.05$ are considered significant.
